# Maternal Food Provisioning in a Substrate-Brooding African Cichlid

**DOI:** 10.1371/journal.pone.0099094

**Published:** 2014-06-09

**Authors:** Kazutaka Ota, Masanori Kohda

**Affiliations:** Department of Biology and Geosciences, Osaka City University, Sumiyoshi, Osaka, Japan; University of Basel, Switzerland

## Abstract

Fish demonstrate the greatest variety of parental care strategies within the animal kingdom. Fish parents seldom provision food for offspring, with some exceptions predominantly found in substrate-brooding Central American cichlids and mouth-brooding African cichlids. Here, we provide the first evidence of food provisioning in a substrate-brooding African cichlid *Neolamprologus mondabu*. This fish is a maternal substrate-brooding cichlid endemic to Lake Tanganyika, and feeds on benthic animals using unique techniques–individuals typically feed on the surface of sandy substrates, but also expose prey by digging up substrates with vigorous wriggling of their body and fins. Young also feed on benthos on the substrate surface, but only using the first technique. We observed that feeding induced by digging accounted for 30% of total feeding bouts in adult females, demonstrating that digging is an important foraging tactic. However, parental females fed less frequently after digging than non-parental females, although both females stayed in pits created by digging for approximately 30 s. Instead, young gathered in the pit and fed intensively, suggesting that parental females provision food for young by means of digging. We tested this hypothesis by comparing the feeding frequency of young before and after digging that was simulated by hand, and observed that young doubled their feeding frequency after the simulated digging. This suggests that parental females engage in digging to uncover food items that are otherwise unavailable to young, and provision food for them at the expense of their own foraging. This behavior was similar to what has been observed in Central American cichlids.

## Introduction

Organisms experience heightened vulnerability to threats such as disease and predation, early in their life history [1], [2]. Many species have evolved parental care strategies to increase offspring survival during this period [3]–[5]. In general, parental care includes preparation of a nest, egg care (e.g. incubation and fanning), brood guarding and nourishment. Parental nourishment largely influences the early growth and development of offspring, and thus their survivorship and future reproductive performance [6]–[9]. Lecithotrophy (yolk-only provisioning) is the most prevalent form of parental nourishment among oviparous species [10]. In addition, parents may provide nutrition to their offspring even after hatching, as offspring have not developed traits useful to search, detect, and handle food; this is especially prevalent in mammals, many altricial birds and some insects [3].

Teleost fish demonstrate the greatest variety of parental care among the animal kingdom, although parental care is typically uncommon [11]–[13]. Fish parents generally do not provision nutrition to offspring, other than yolk, but a handful of exceptions mainly exist in cichlids that show an unrivalled diverse array of parental care strategies [14]–[16]. For example, in the mouth-brooding African cichlids *Tropheus duboisi*, *T*. *moorii* [17] and *Cyphotilapia frontosa* [18], part or all of the food taken by female parents are ingested by their young held in their buccal cavity. In the substrate-brooding Central American cichlids *Symphysodon discus* [19] and *Amphilophus citrinellus* (formerly *Cichlasoma citrinellum*) [20], young feed on epidermal mucus on parents’ bodies. Furthermore, in other substrate-brooding Central American cichlids, *Amatitlania siquia* (formerly *Archocentrus nigrofasciatum*) [21] and *Rocio octofasciata* (formerly *Archocentrus octofasciatum*) [22], parents increase food availability for their offspring by means of fin digging, where they stir up loose substrate with a short bout of vigorous, rapid beating of their pectoral fins.

The substrate-brooding African cichlid *Neolamprologus mondabu* is known to engage in fin-digging for its own feeding [23]. This cichlid is endemic to Lake Tanganyika in East Africa, and preferentially inhabits sandy substrates in rocky areas, where it feeds on benthic animals [24]. Its prey occur within the substrates as well as on the substrate surface, and *N*. *mondabu* feed using the following four methods [23]: (1) picking–moving slowly or darting a short distance and picking up prey; (2) thrusting–sucking prey while thrusting the mouth into loose substrate with a short dash, then ejecting inedible matter through the gills and mouth; (3) flipping–picking up prey exposed by lifting and flipping over pebbles or small vacant shells; and (4) digging–intensively feeding on prey exposed in pits that are dug in loose substrate using vigorous wriggling of the body, pectoral fin and caudal fin. Fry spread horizontally around nest burrows and peck at substrate to feed on the similar benthic animals as their parents, although they also feed on plankton in the water column in earlier postlarval stages [25], [26]. Fry do not use digging and therefore have no access to benthic animals that remain in the substrate and under pebbles. We witnessed several events during which parental females engaged in digging, but appeared to feed less frequently in the fin-dug pits while their young gathered and foraged there. We propose that parental females are provisioning food for their offspring through digging. If this is the case, we predicted that parental females would demonstrate a reduced feeding frequency, and that young would increase their feeding frequency following parental digging. We tested these predictions in the field through observations and experimental manipulations.

## Materials and Methods

### Study Species

Both sexes of adult *N*. *mondabu* (>60 mm standard length [SL]) defend territories against same-sex rivals and food competitors [27]. Males are polygynous and their territories encompass 1–6 female territories [27], [28]. Spawning takes place in a burrow that the female digs under a rock within her territory. Females care for eggs and embryos in the nest burrows and subsequently guard free-swimming young until independence at *ca*. 10 weeks of age, during which time the young grow rapidly [29]. Females spawn 140 eggs on average, but the survival rate is considerably low among Lake Tanganyika cichlids [29]. Males pay no regard to their offspring while periodically visiting female territories [27], [28].

### Field Study

To test our hypotheses, field surveys were conducted in September 2013 at depths of 4–10 m at Nkumbula Island (8°75′ S, 31°09′ E), near Mpulungu, Zambia. Although *N*. *mondabu* is common in the littoral zone, brood-guarding females are present at a low density (0.002–0.004/m^2^ [Ota unpublished data]). To quantify female feeding behaviors, we looked for parental females in a large area (*ca*. 5000 m^2^) and non-parental females within a subset (*ca*. 900 m^2^); the observation area consisted of rocks and sandy substrate that this fish prefers. We found a total of 17 females (*n*_non-parental_ = 8, *n*_parental_ = 9) and recorded the behaviors of each fish for 30 min (two 15-min periods in a row) using a digital camera. Pecking at the substrate or rock surface was classified as a feeding bout, but we could not monitor all feeding events within the 30 min as individuals occasionally moved under or behind rocks (range: 0–384 s, *n* = 17). Counts of feeding events were therefore limited to unobstructed 15-min periods. However, visual obstruction did not affect our count of digging events because individuals have insufficient space to dig when under rocks. Therefore, we counted the number of digging events within 30 min and recorded the amount of time that females spent in the dug pit after each digging event. We observed 65 digging events from 17 females during the observations. To examine the effect of brood care on feeding, we also counted the number of attacks against approaching fish for unobstructed 15 min.

To examine the effect of digging on feeding by young, we compared their feeding frequency between before- and after-digging periods using a repeated-measures design. Since digging is infrequent and unpredictable, spontaneous digging could not be used to collect sufficient samples. Instead, we simulated digging by quivering a hand for approximately 2 s, and compared the feeding frequency of young before and after digging. We performed this experiment with 11 clutches; three young from each clutch were randomly selected. To avoid cofounding observations of individual offspring feeding before and after simulated digging, we performed manipulations separately for each young; we counted the feeding bouts of an individual young over five min, then simulated digging near it and counted feeding events within the next five min. The latter observations began after 30 s of digging. The simulated digging successfully induced feeding in the dug pits, although young usually fled from the hand briefly before returning. To avoid multiple observations of the same individuals, the first young was captured immediately following post-digging observations and observations of the third individual began immediately following the second individual’s experiment. Captured young were held in a plastic bag and released at the end of the experiment.

Our study complied with the current laws of Zambia and Japan, and was approved by the Zambian Ministry of Agriculture, Food and Fisheries for fish research in Lake Tanganyika.

### Statistical Analysis

The frequencies of attacks, feeding and digging were compared between parental and non-parental females using generalized linear models (GLMs). Because digging was measured repeatedly in some females, time spent in dug pits, the number of feeding events in pits and frequency of feeding were compared using generalized linear mixed models (GLMMs); a female identifier included as a random factor. The feeding frequency of young before and after simulated digging was compared using a GLMM with two random factors (young and clutch identifiers) included. Difference in stages of young was not considered because of inadequate sample size. We fitted a Gaussian distribution to the models when data were normally distributed, while other count and frequency data were fitted to a Poisson or negative binomial (if overdispersion was observed) distribution. All analyses were performed using R version 2.15.2.

## Results

Non-parental females were observed feeding much more frequently than parental females for15 min (Table 1). In non-parental females, feeding induced by digging accounted for 29.5% (mean; SD = 19.5, range: 3.8–63.9%, *n* = 8) of total feeding bouts; although the success of every feeding bout could not be confirmed, this indicates that digging was a significant behavior for obtaining nutrition. Parental and non-parental females spent similar amounts of time following digging in the fin-dug pit, but parental females fed less frequently during the period than non-parental females (Table 1, see Movies S1 and S2). Parental females performed digging marginally less frequently than non-parental females, but practiced aggressive attacks more frequently than non-parental females (Table 1). There was a tendency of negative correlation between these behaviors across females (negative binomial GLM, *χ* ^2^ = 3.01, *df* = 1, *p* = 0.08).

**Table 1:** Differences in feeding activities between parental and non-parental *N*. *Mondabu* females.

			statistics		
Variables	parental females	non-parental females	*F*/*χ* ^2^	*df*	*p*
Number of attacks (/15min)†	14.7±9.5 (9)	7.4±4.5 (8)	3.92	1,15	0.066
Number of feedings (/15min)†	76.4±31.2 (9)	134.4±40.5 (8)	11.01	1,15	0.005
Time spent in dug pit after diggings (sec)^‡^	28.2±21.2 (9)	32.0±14.4 (8)	1.18	1	0.278
Number of feeding in dug pit after digging^§^	4.7±2.4 (9)	12.7±7.4 (8)	4.70	1	0.030
Frequency of feeding in dug pit after digging (/sec)^§^	0.20±0.13 (9)	0.36±0.07 (8)	9.04	1	0.002
Number of digging (/12min)^||^	2.7±2.6 (9)	5.1±4.7 (8)	2.50	1	0.11

† LM; ^‡^GLMM; ^§^Poisson GLMM; ^||^negative binomial GLM.

Values are means ± SD. Sample sizes are in parentheses.

**Figure 1:**
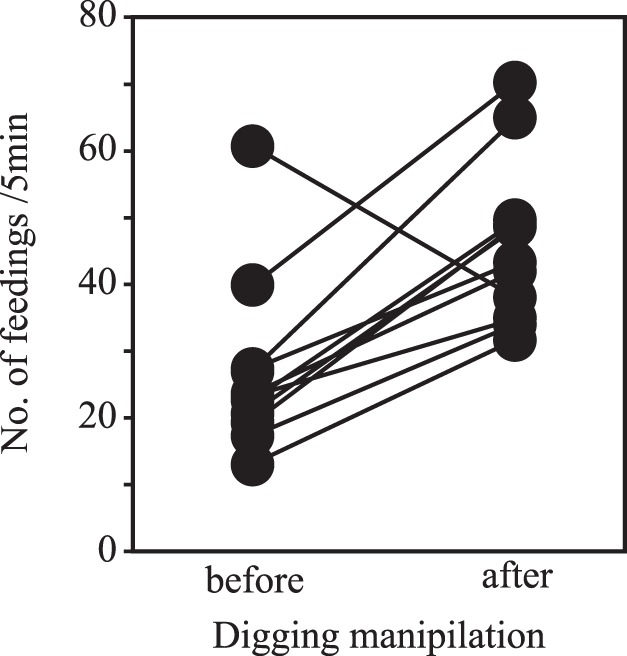
Differences in feeding frequency by young between periods before and after hand-simulated digging. Each plot is a mean value in each clutch.

Free-swimming young foraged at a mean frequency of 26.8 times per 5 min (SD = 14.9, *n* = 33 young). They gathered in the hand-dug pits within 38 s following simulated digging, on average (SD = 45.7, *n* = 33), and doubled their frequency of feeding during the 5 min following digging compared to their feeding frequency during the 5 min before digging (GLMM, *F = *39.07, *df* = 122.8, *p*<0.001; Fig. 1).

## Discussion

Non-parental females relied on digging for 30% of total feeding bouts. Conversely, parental females marginally decreased digging frequency and significantly decreased the frequency of feeding following digging, compared to non-parental females. We found a negative correlation between the numbers of digging behaviors and aggressive attacks, suggesting that the decrease in digging frequency is attributable to an increase in vigilance for brood defense. However, brood defense is an unlikely cause for the decreased feeding observed after digging because the time spent in the dug pit did not differ between parental and non-parental females. Our experiment showed that young increased their feeding frequency after hand-simulating digging. These results suggest that parental females sacrifice their own foraging opportunity to provision food for their young by digging to uncover food items that would otherwise be unavailable to them. Young foraged twice as much after parental digging relative to normal foraging, suggesting that food provisioning contributed considerably to their nourishment. The contribution from parental digging would be actually much greater than the current estimate from our simulated-digging experiment, given that young immediately enter pits dug by parental females or sometimes gather around females prior to digging but did not necessarily rush into pits immediately following simulated digging. The improved accessibility to food would enhance the growth rate of young, and thus contribute to increased female fitness. For example, an increase in growth rate would increase size at independence, and thus survival of young due to increase in ability to escape predation and future reproductive performance [6]–[9], [22], [30]–[32]. Enhanced growth rate would also decrease the time required to reach independence, which may enable females to shorten the interval to the next mating [32]–[34]. In a mouth-brooding Lake Tanganyika cichlid *Tropheus moorii*, young that are provisioned by parents grow larger, and thus have superior competitive and predator-avoidance abilities, compared to unprovisioned young [35]. Increased growth through improved accessibility to food by parents would be beneficial for *N*. *mondabu*, especially considering young of this fish experience high mortality rates compared to other Lake Tanganyika cichlids [29].

To our knowledge, this is the first study to show parental food provisioning in substrate-brooding African cichlids. Why does only *N*. *mondabu* provision food for its offspring among them? The reason is probably related to limited access to food of their young. In fish, young are generally capable of feeding after yolk sac absorption (i.e., precocial). For example, young of many substrate-brooding Lake Tanganyika cichlids begin feeding on plankton immediately after yolk sac absorption under parental care, and access to food is not restricted by their own abilities. However, young *N*. *mondabu* feed on benthos, as adult *N*. *Mondabu* do, but only by means of picking because of insignificant power to dig and expose prey; they only supplement their diet with plankton in earlier postlarval stages. This suggests that food accessibility in this fish is limited by individuals’ abilities, and thus their growth may be highly restricted without parental provisioning. Likewise, parents in some mouth-brooding African cichlids provision food for their young that are mainly held in females’ buccal cavities, and thus have limited access to food [17], [18], [35]. In addition, female territoriality is likely also partly responsible for the limited access to food by young. The prey of benthivorous *N*. *mondabu* are distributed discretely on substrate, and females are highly aggressive against both conspecific and heterospecific food competitors to defend feeding territories [27]. The energetic demand of these defensive behaviors would require increased foraging within territories, likely at the expense of constant care and attention to their young. Feeding on plankton in predator-vulnerable water columns should be risky for young under loose parental care, and as such, feeding in the water column is limited to the earlier postlarval stages. Food provisioning may be a compromise between parental females’ own feeding and parental care.

Food provisioning is expected to impose costs on parental female *N*. *mondabu* because they partly overlap food items with their own young in their territories [29], and they sacrifice their own feeding for provisioning offspring. Therefore, parents and young are expected to be in conflict [32]; we observed that young typically rush in whenever parental females approach nest burrows, but the females do not always respond (Ota pers. obs.). We do not know whether or how young actively solicit food provisioning from their parent or what motivates parents to provision food. In Central American cichlids, parents provide food to their offspring according to the parents’ saturation status [21], [22]. Parents may also be motivated to provision in response to behavioral cues from young, such as rushing by young (e.g., begging in bird species [36]) or chemical cues, such as odors concentrated by aggregating young (e.g., chemical solicitation in insect species [37]). Further studies will be conducted to examine the optimization of provisioning and solicitation strategies.

This study offers one of the few scarce examples in parental care in fishes where parents seldom nourish their offspring after hatching. Notably and surprisingly, the form of food provisioning in *N*. *mondabu* is similar to what is observed in substrate-breeding Central American cichlids, whereby both sexes (but more frequently females) engage in digging and provisioning [15], [16]. Given the phylogenetic and biogeographic distances between African and Central American cichlids [16], [38] and the lack of food provisioning in most other cichlids, these similar forms of food provisioning should evolved independently.

## Supporting Information

Movie S1 **Non-parental female digging.** Females are observed intensively feeding on the freshly dug pit.
(WMV)

Movie S2 **Parental female digging.** Young are observed rushing toward their mother as she digs, and intensively feeding in the freshly dug pit.
(WMV)
